# Sustainable Chromium (VI) Removal from Contaminated Groundwater Using Nano-Magnetite-Modified Biochar via Rapid Microwave Synthesis

**DOI:** 10.3390/molecules26010103

**Published:** 2020-12-28

**Authors:** Xiaoming Song, Yuewen Zhang, Nan Cao, Dong Sun, Zhipeng Zhang, Yunlong Wang, Yujuan Wen, Yuesuo Yang, Tao Lyu

**Affiliations:** 1 Key Lab of Regional Environment and Eco-Restoration, Shenyang University, Ministry of Education, Shenyang 110044, China; songxm@syu.edu.cn (X.S.); yuewenzhang12@163.com (Y.Z.); wyldlnu@163.com (Y.W.); yujuanwen@syu.edu.cn (Y.W.); 2 Chengdu Center of Hydrogeology and Engineering Geology, Sichuan Bureau of Geology & Mineral Resources, Chengdu 610081, China; caonan@schuadi.com (N.C.); sundong@schuadi.com (D.S.); Zhangzhipeng@schuadi.com (Z.Z.); 3 Key Lab of Groundwater Resources and Environment, Jilin University, Ministry of Education, Changchun 130021, China; 4 Cranfield Water Science Institute, Cranfield University, College Road, Cranfield, Bedfordshire MK43 0AL, UK

**Keywords:** nano-magnetite, biochar, adsorption kinetics, heavy metal, thermodynamics

## Abstract

This study developed a nano-magnetite-modified biochar material (m-biochar) using a simple and rapid in situ synthesis method via microwave treatment, and systematically investigated the removal capability and mechanism of chromium (VI) by this m-biochar from contaminated groundwater. The m-biochar was fabricated from reed residues and magnetically modified by nano-Fe_3_O_4_. The results from scanning electron microscopy (SEM) and X-ray diffraction (XRD) characterisations confirmed the successful doping of nano-Fe_3_O_4_ on the biochar with an improved porous structure. The synthesised m-biochar exhibited significantly higher maximum adsorption capacity of 9.92 mg/g compared with that (8.03 mg/g) of the pristine biochar. The adsorption kinetics followed the pseudo-second-order model and the intraparticle diffusion model, which indicated that the overall adsorption rate of Cr(VI) was governed by the processes of chemical adsorption, liquid film diffusion and intramolecular diffusion. The increasing of the pH from 3 to 11 significantly affected the Cr(VI) adsorption, where the capabilities decreased from 9.92 mg/g to 0.435 mg/g and 8.03 mg/g to 0.095 mg/g for the m-biochar and pristine biochar, respectively. Moreover, the adsorption mechanisms of Cr(VI) by m-biochar were evaluated and confirmed to include the pathways of electrostatic adsorption, reduction and complexation. This study highlighted an effective synthesis method to prepare a superior Cr(VI) adsorbent, which could contribute to the effective remediation of heavy metal contaminations in the groundwater.

## 1. Introduction

Chromium (Cr) is a common groundwater contaminant at hazardous sites, which could be released from intense anthropogenic activities, including chemical synthesis, mining, dyeing, metallurgy and wood preserving industries [1,2]. Cr appears most commonly as Cr(VI) and Cr(III) in aqueous solutions [3]. Cr(VI) is highly mobile in water, which could cause an acutely toxic and carcinogenic effect to humans, animals, plants and microorganisms [4,5]. Cr(III), by contrast, is less toxic and could precipitate in the aquifer in the form of oxides or hydroxides with poor migration ability [6]. Therefore, the reduction of Cr(VI) to Cr(III) has been deemed as a key process for the treatment/detoxification of Cr-contaminated waters [7]. Cr(VI) in the groundwater is difficult to be naturally converted into Cr(III) because of the lack of electron donors, such as carbon sources [8]. Given the long-term storage characteristic, the technique development on effective Cr(VI) removal from polluted groundwater has become urgently important.

Various technologies, such as electrodynamic methods, chemical reduction methods and biological remediation methods, have been deployed for the treatment of Cr(VI) from groundwater [9]. Among these practised techniques, physical adsorption has recently attracted much attention because of its characteristics of easy operation, low cost and reduced secondary pollution potential [10]. Moreover, the adsorbents could be easily reused after a proper desorption treatment. Biochar, derived from biomass waste, has been largely used for the removal of heavy metals, including Cr, from the wastewater attribute to its large specific surface and inherent porous structures [11]. Further efforts have been made to increase the adsorption efficiency through increasing the active adsorption sites in the biochar surface [11]. Yu et al. (2020) have modified the corn stalks biochar with ZnCl_2_, and achieved 61.67% improvement of the Cr(VI) adsorption capacity from wastewater [12]. Additionally, current research also focuses on technology development to the separation of the tiny biochar particles from the treated solutions.

The magnetic separation has been proven as a selective and efficient approach of solid-liquid separation, compared with traditional methods of centrifugation, filtration, and sedimentation [13]. Following this concept, introducing the magnetic medium (e.g., zero-valent iron (ZVI), and magnetite (Fe_3_O_4_)) to the biochar through chemical co-precipitation has been developed to enable the magnetic biochar to be effectively separated from the solution via magnetic separating techniques [14]. More importantly, previous studies have demonstrated that the modified magnetic biochar could also significantly improve the adsorption rate of Cr(VI) from 26.3% to 83.5% because of the increased adsorption sites of the adsorbent [13,15,16]. Nevertheless, current modification/synthesis processes are generally time-consuming (>hours) and high-energy needs (>500 °C) [17,18,19]. For example, Liyanage et al. (2020) and Karunanayake et al. (2017) synthesised the magnetic biochar through adding FeCl_3_ and FeSO_4_·7H_2_O, where the reactions were conducted under 900–1000 °C for 2 h [16,20]. Han et al. (2016) prepared magnetic peanut hull-derived biochar through pyrolysis treatment at 650 °C for 1 h [21]. The pyrolysis method was also used by Yang et al. (2016) for magnetic biochar preparation at various temperatures of 500 °C, 600 °C, 700 °C and 800 °C [22]. Thus, it is urgently needed to develop an effective and cost-saving method for magnetic biochar preparation. Because magnetite is widespread in the earth’s crust and iron is one of the main components, moreover, the application of ferrous nanomaterials in the remediation of water pollution could avoid secondary pollution. Plant-biomass derived biochar is an eco-friendly and green material, which has been used frequently as a sustainable bio-adsorbent to remove toxic metals or soil amendment [23]. Therefore, nano-magnetite modified biochar has a broad application prospect in the remediation of groundwater pollution.

In this study, Cr(VI) removal performances, kinetics and isotherms by the nano-magnetite-modified biochar (m-biochar) via rapid microwave treatment were evaluated to compare with the performances of the pristine biochar. The effects of pH conditions (3–11) and ionic strength on the Cr(VI) adsorption were also investigated. X-ray diffraction (XRD), Fourier transform infrared spectroscopy (FTIR), scanning electron microscopy (SEM), energy-dispersive X-ray spectroscopy (EDS) and X-ray photoelectron spectroscopy (XPS) detection technologies were used to reveal the potential mechanisms of the Cr(VI) adsorption process. The developed m-biochar is proposed to be used in permeable reactive barriers (PRB) for the remediation of Cr(VI) contaminated groundwater. Because of the challenge to recycle the M-biochar in the actual application of PRB, further studies will be carried out to evaluate the recyclability of the material. The main objective of this study is to synthesize an eco-friendly nano-magnetite-modified biochar (m-biochar) material in order to efficiently and sustainably remove Cr(VI) from the contaminated groundwater. The effort is also made to develop and evaluate a simple and rapid in situ method for the preparation of the material. The results of the study could provide evidence-based insights of using cost-effective green nano-technology for the remediation of heavy metal contaminated groundwater.

## 2. Results and Discussion

### 2.1. Characterisation of the Pristine and Nano-Magnetite Modified Biochar

**Figure 1:**
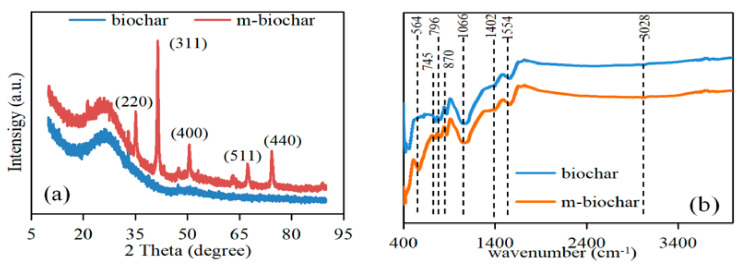
XRD (**a**) and FTIR (**b**) patterns of the pristine biochar and m-biochar.

XRD analysis was conducted in order to identify the compositions of the reed-derived biochar before and after the magnetic modification. The XRD pattern of m-biochar is identical to magnetite (Figure 1a). The representative diffraction peaks at the positions of 35.2°, 41.5°, 50.6°, 67.4° and 74.4° matched well with the standard PDF card of Fe_3_O_4_ (PDF#75-1610). Such diffraction peaks were also quantified by the previous study of synthesised magnetic biochar by zero-valent iron through co-pyrolysis method [24]. The results confirmed that Fe_3_O_4_ was successfully doped onto the biochar material.

**Figure 2:**
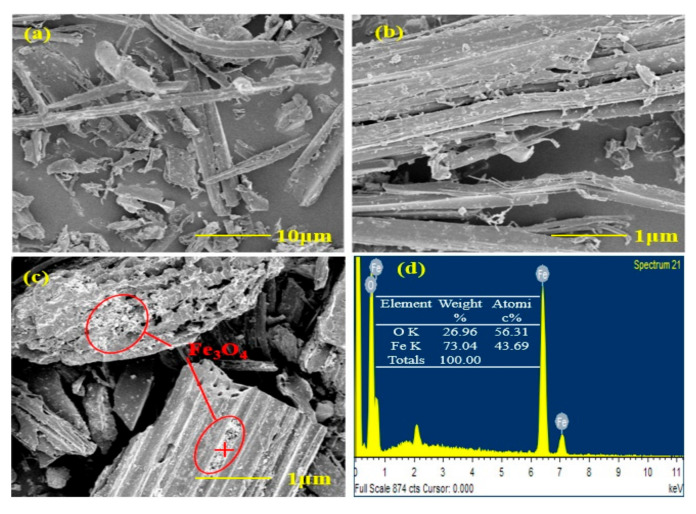
SEM images and EDS spectra of the pristine biochar and m-biochar: (**a**,**b**) are the SEM images of the pristine biochar; (**c**,**d**) are the SEM images and EDS spectra of m-biochar.

From the FTIR spectra, there are a few peaks that appeared for both pristine biochar and m-biochar (Figure 1b). The peaks at 870, 796 and 745 cm^−1^ were characteristic peaks of the C-H bond [25]. The peak at 1066 cm^−1^ was the C-O stretching vibration in the structure of aromatic ether, carbohydrate, or polysaccharide. The peaks near 1402, 1554 and 3028 cm^−1^ were the stretching vibration peaks of C=O, C=C, and of C-H on the aromatic ring [26]. The results agreed with the biochar characteristics that not only contain porous structure but also numerous active adsorption sites for Cr removal [12]. Moreover, a distinct peak at 564 cm^−1^ was found for m-biochar (Figure 1b), which was the characteristic stretching peak of Fe-O [27]. The results demonstrated magnetic modification increased the species and number of functional groups of the biochar, which may contribute to the Cr (VI) adsorption. Based on the images from the SEM analysis (Figure 2), the pristine biochar was stripped after carbonisation at high temperature, which resulted in a rough and relevant random distributed porous structure (Figure 2a,b). However, the surface morphology of m-biochar was significantly different compared to the pristine biochar (Figure 2c). The pore structure on the surface was more developed and the surface roughness was increased. Therefore, its specific surface area and pore volume (Table 1) were higher than those of the pristine biochar. In addition, nano-scale spherical particles of polymer were observed on the surface of m-biochar, which was further detected with EDS to quantify its composition (Figure 2d). The mass fraction of Fe in the m-biochar was 6.16%. The high composition of Fe_3_O_4_ further proved the success of the magnetic modification of biochar, which agreed with the XRD and FTIR characterisations.

### 2.2. Adsorption Performances and Kinetics

**Figure 3:**
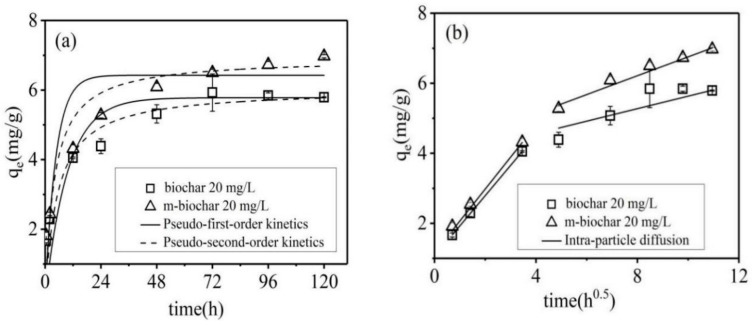
The kinetics of Cr(VI) adsorption on the pristine biochar and m-biochar and the simulated pseudo-first-order kinetics and pseudo-second-order kinetics models (**a**) and the intra-particle diffusion model (**b**).

Cr(VI) adsorption performances and kinetics of both pristine biochar and m-biochar were investigated under different initial Cr(VI) concentrations, i.e., 20, 40 and 80 mg/L (Table 2). Both adsorbents showed rapid adsorption rates during the initial stage within 12 h, and reached 60% of the equilibrium adsorption amount. Then, the adsorption rates subsequently slowed down until the equilibrium was reached by around 48 h under initial Cr(VI) concentration of 20 mg/L (Figure 3a). The Cr(VI) adsorption dynamics under other initial concentrations showed the same tendency (data is not shown). In the study of Kumari et al. (2015), the adsorption process of Cr(VI) showed a fast adsorption process in the first 1 h, which removed 44% of the Cr(VI), and the equilibrium was reached after 48 h [15]. Other studies also found a similar tendency of Cr(VI) adsorption, however, with various stabilisation times of 18 h [28], 25 h [29] and 96 h [30]. The variance of the equilibrium time is expected to heavily depend on the characteristics of the adsorbents, such as the existence of different acidic and alkaline functional groups on their surfaces. In this study, m-biochar showed clearly fast adsorption speed and higher equilibrium values compared with those of the pristine biochar (Figure 3a). It may mainly attribute to the existence of active Fe-O sites (Figure 1) for Cr(VI) adsorption and more developed pore structure (Figure 2).

Pseudo-first order model and pseudo-second-order model were initially applied to simulate the kinetic data (Table 2). The adsorption processes of both adsorbents under all initial Cr(VI) concentrations were better fitted to the pseudo-second-order model (*R*^2^ > 0.92), which supported the premise that adsorption process was dominated by the chemisorption [2,14]. The theoretical equilibrium adsorption amount of Cr(VI) obtained by fitting the pseudo-second-order kinetic equation was consistent with the experimentally measured equilibrium adsorption amount. Moreover, the adsorption capacity of Cr(VI) on m-biochar (6.22–7.46 mg/g) was significantly higher than that (5.55–6.94 mg/g) of pristine biochar.

To further understand the diffusion steps of the Cr(VI) adsorption, the intraparticle diffusion model (Weber-Morris Model) was applied to fit the kinetic data (Figure 3b, Table 2). The results indicated that, besides chemisorption, diffusion processes, especially intraparticle diffusion, may also affect the Cr(VI) adsorption rate on the solid solution interface. Data from the two materials could be divided into two segments, where the plot portions could be attributed to bulk diffusion, intra-particle diffusion until equilibrium [31]. The diffusion rate constants k_d1_ > k_d2_ and the boundary layer C_1_ < C_2_ indicated that the diffusion rate of Cr(VI) on the surface of the material was faster, while the diffusion rate within the particles was relatively slow [32]. It may be caused by the decrease of Cr(VI) concentration after the rapid diffusion of the boundary layer after the initial stage of adsorption [33]. The adsorption rate of Cr(VI) by the two materials was mainly controlled by the intra-particle diffusion until final equilibrium is reached [34]. The results supported that the Cr(VI) adsorption rate by both biochar materials was controlled by the boundary layer diffusion and intra-particle diffusion.

### 2.3. Adsorption Isotherm

**Figure 4:**
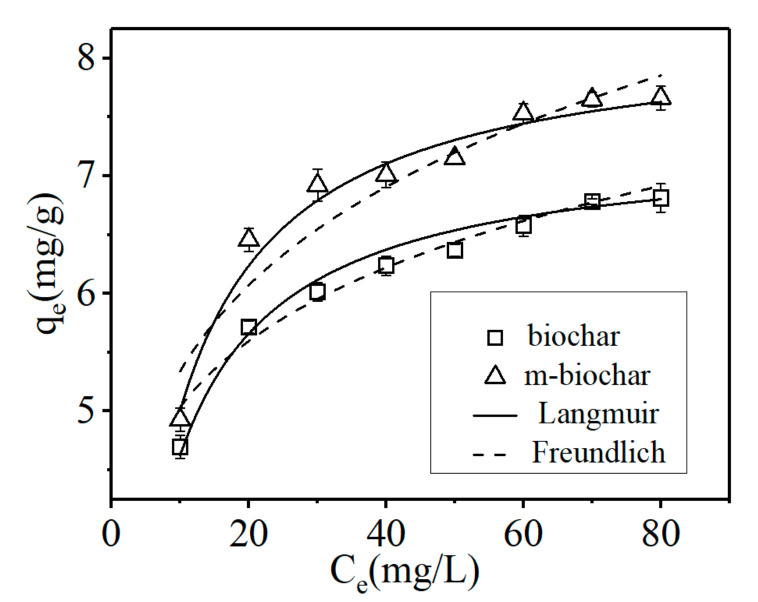
Langmuir and Freundlich nonlinear plots of adsorption isotherms for Cr(VI). The error bars represented the standard deviation.

An adsorption isotherm study was conducted in order to evaluate the Cr(VI) adsorption capabilities of the pristine biochar and m-biochar. Langmuir and Freundlich models were applied separately to simulate the experimental data. As illustrated in Figure 4, the Freundlich isotherm model exhibited slightly better fitting results (*R*^2^ > 0.92) than the Langmuir model (*R*^2^ > 0.90, Table 3). According to the Freundlich model, the Cr(VI) adsorption capacity of m-biochar was significantly higher than that of the pristine biochar. It may be attributed to the active Fe-O sites of m-biochar and increased specific surface area and the pore structure during the magnetisation process (Table 1). In addition, the high ion exchange capacity of the microporous surface of the biochar and a large number of oxygen-containing functional groups could significantly enhance the adsorption of heavy metal ions [35]. The magnetic modification increased the species and number of functional groups of the biochar and it has more active Fe-O sites, which may contribute to the Cr(VI) adsorption. Moreover, the positive charge on the surface of m-biochar increased greatly after modification which could increase the interaction with the chromium anion [36].

### 2.4. Effect of pH and Ionic Strength on Cr (VI) Adsorption

Cr(VI)-contaminated real wastewater may contain a high range of pH, which could affect the behaviour of functional groups on the surface of biochar and influence the Cr(VI) adsorption capabilities [37,38]. The zeta potentials of the pristine biochar and m-biochar both decreased along with the increase of groundwater pH from 3 to 11 (Table 4). Under the acidic condition, the zeta potential of both biochar was positive, and the m-biochar showed significantly higher values (19.7–2.7) than those (3.72–7.99) of pristine biochar. In the neutral and alkaline conditions, the zeta potentials of both biochar materials became negative. The data summarised from previous studies are listed in Table 4, which also support this negative correlation between the aqueous pH and the zeta potentials of different biochar materials.

**Figure 5:**
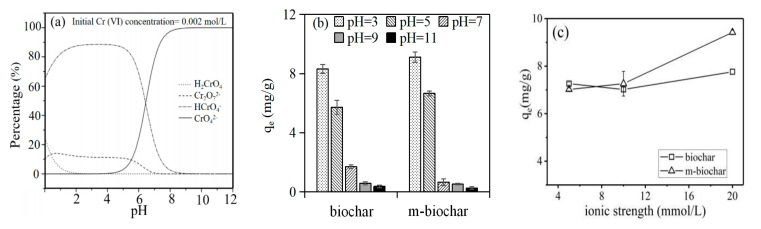
(**a**) The chromium ion species in the groundwater under different pH conditions (the modified figure was adapted from Zhou et al, 2016) [37] (**b**) effects of pH on Cr(VI) adsorption, and (**c**) effects of ionic strength on Cr(VI) adsorption.

Cr(VI) in groundwater mainly existed as anionic forms, such as CrO_4_^2−^, HCrO_4_^−^ and Cr_2_O_7_^2−^. It is reported that HCrO_4_^−^ and CrO_4_^2−^ were the main forms of Cr(VI) when the solution pH was below and above 6.5, respectively (Figure 5a). Moreover, if the total chromium concentration was above 1 g/L under acidic condition, Cr_2_O_7_^2−^ would be the dominated forms [43]. Therefore, adsorbents with a positive charge (higher zeta potential) could benefit adsorbing Cr(VI) anions through the electrostatic attraction [44]. Under neutral and alkaline conditions, biochar with negatively charged, an electrostatic repulsion would be formed between the absorbents and negatively charged Cr(VI) and reduced the ability to adsorb Cr(VI).

In this study, both pristine biochar (8.03 mg/g) and m-biochar (9.92 mg/g) showed highest Cr(VI) adsorption under pH of 3, compared with the abilities under higher pH conditions (Figure 5b). The adsorption capacities decreased to 0.095 and 0.435 mg/g along with the increasing initial pH until 11 for pristine biochar and m-biochar, respectively. Since the zeta potential of m-biochar was higher than that of biochar under the same pH condition (Table 4), m-biochar always showed clearly higher Cr(VI) adsorption capability (0.435–9.92 mg/g) compared to those (0.095–8.03 mg/g) of the pristine biochar. In addition, the specific surface area of the m-biochar was three times larger than that of the biochar, but the maximum adsorption capacity of Cr(VI) did not increase proportionally to the surface area under the same pH condition. It may be because the electrostatic interaction plays a dominated role in the adsorption of Cr(VI) on m-biochar rather than the surface adsorption and micropore diffusion. Kumari et al. (2015) have reported that the optimum pH for Cr(VI) adsorption was 4.0 [15], the removal rate of Cr(VI) could decrease 3 to 7 times when the pH increased from 3 to 4.5 [45]. The previous study also proved the same tendency for the Fe_3_O_4_ modifier on Cr(VI) adsorption, where the adsorption capacity of mesoporous magnetite (Fe_3_O_4_) nanospheres to Cr(VI) decreased gradually when pH increased from 2 to 7 [15]. The theories support the results from the current study that the significant lower Cr(VI) adsorption was found along with the increasing pH conditions (Figure 5b).

Cr(VI) adsorption capabilities of both pristine biochar and m-biochar were investigated under different ionic strength (i.e., 5, 10 and 20 mmol/L of NaCl addition). Increasing ionic strength had little effect on Cr(VI) adsorption by biochar, where the corresponding adsorption capacities of Cr(VI) stayed in the range between 7.02 and 7.76 mg/g (Figure 5c). However, the adsorption capacity of Cr(VI) by m-biochar significantly increased from 7.06 to 9.42 mg/g) along with the increasing of the ionic strength. It was supported by Pham et. al., 2015 [46], the ionic strength would become the crucial factor and positively correlated with the Cr(VI) adsorption when the electrostatic interaction dominates as adsorption mechanisms.

### 2.5. Cr Adsorption Mechanism by the Nano-Magnetite-Modified Biochar (m-Biochar)

**Figure 6:**
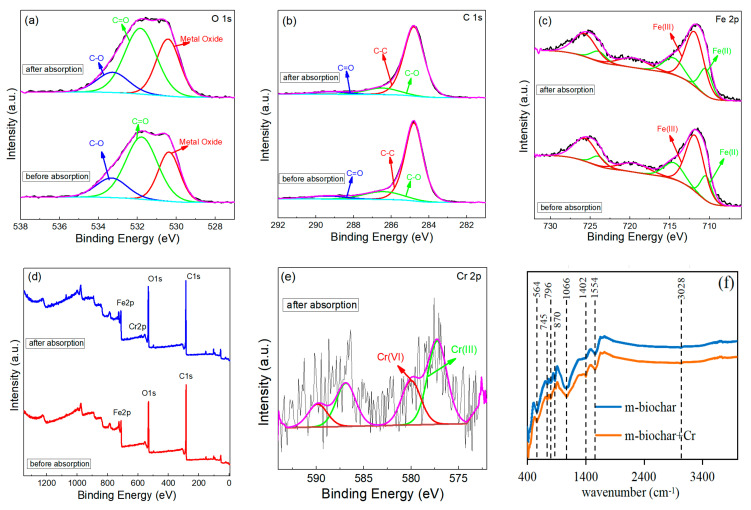
XPS and FTIR spectra of m-biochar before and after Cr(VI) adsorption: the narrow scan of O 1s (**a**), C 1s (**b**), Fe 2p (**c**) spectra, wide-scan survey (**d**), Cr 2p spectra (**e**), and FTIR spectra (**f**).

The FTIR and XPS analysis were carried out to detect the m-biochar after the adsorption in order to reveal the Cr(VI) adsorption mechanisms (Figure 6). The previous discussions demonstrated that Cr(VI) was likely adsorbed on nano-Fe_3_O_4_ of m-biochar through electrostatic interaction. The FTIR spectrum of the m-biochar after the adsorption experiment showed weakened C-H characteristic peaks at 870 cm^−1^ and strengthened C=O functional groups at 1402 cm^−1^ (Figure 6f), which may due the high oxidising ability of Cr(VI) and oxidise the -CH bond to -COO^−^ bond [47]. These functional groups are reductive, however, as shown in the FTIR spectrum, they rarely participated in the reduction reactions [19]. The total area of O 1s peaks increased after the Cr(V) adsorption (Figure 6a), and the atomic ratio of metal oxide increased from 7.83% to 10.21%, which may be caused by the occurrence of Cr-O bond [19].

Fe_3_O_4_ contains Fe(II) and Fe(III) and may provide the lattice oxygen in metal oxides as Fe_3_O_4_ (532 eV), FeO (531.99 eV) and Fe_2_O_3_ (532.5 eV) [17,22]. After the completion of adsorption, the peak of Fe(II) decreased from 2.21% to 1.91% (Figure 6c). The change may be caused along with the Cr(VI) reduction by Fe(II), such redox reaction may lead to a certain amount of Cr(III) generation [13]. The reaction processed can be explained in Equations (1) and (2) as follows:

(1) $$Cr(VI)+Fe_{3}O_{4}(FeO)\to FeCr_{2}O_{4}+Fe_{2}O_{3}$$

(2) $$(1-x)Cr(III)+xFe(III)+3H_{2}O\to Cr_{1-x}Fe_{x}{\left(OH\right)}_{3}+3H^{+}$$

After the adsorption of Cr(VI), the peaks of C 1s, O 1s and Fe 2p retained in the m-biochar (Figure 6a–c). However, an extra spectrum of Cr 2p appeared in the material (Figure 6e). The peaks at 580.02 eV and 577.23 eV could be correlated with Cr(VI) and Cr(III), respectively. The existed Cr(III) may be formed as the FeCr_2_O_4_ [48]. Moreover, the ionisable groups, such as hydroxyl (-OH) and carboxyl (-COO^−^), existed on the surface of biochar and could be protonated to form -OH_2_^+^ and -COOH under acidic conditions [49,50]. Thus, the generated -OH_2_^+^ can contribute to the electrostatic adsorption of Cr(VI) [8]. In summary, the adsorption mechanism of Cr(VI) by m-biochar includes electrostatic adsorption, reduction and complexation. Nevertheless, the hypothesis of Cr adsorption mechanisms by the m-biochar requires further investigation and confirmations through more advanced characterisation techniques.

## 3. Materials and Methods

### 3.1. Biochar Materials, Groundwater and Chemicals

The local reed biomass, collected from the bank of the Hunhe River in Shenyang city, Liaoning province, China, was used as the raw material to prepare the biochar. The chemicals, including ferrous sulphate heptahydrate (FeSO_4_·7H_2_O, ≥98%), sodium hydroxide (NaOH, ≥99%), sodium chloride (NaCl, ≥99.5%) and hydrochloric acid (HCl, ≥99.5%), were purchased from Tianjin Damao Chemical Reagent Factory, Tianjin, China. The real shallow groundwater was collected from Qianjin Farm, Shenbei New District, Shenyang China (burial depth less than 20 m). The main components and physicochemical properties of the groundwater have been reported in our previous study [51]. Briefly, the average levels of total dissolved solids and pH of the groundwater were 0.59 g/L and 7.11, respectively. The oxidation-reduction potential (Eh) was within −43.2–80.2 mV. The hardness of groundwater was relatively high, within 354.1–540.2 mg/L (measured with CaCO_3_) with an average of 424.4 mg/L. Moreover, the main cationic components included Ca^2+^, Na^+^, K^+^, Mg^2+^ and some trace elements (e.g., Fe, Mn), and the main anion components were HCO_3_^−^, Cl^−^ and SO_4_^2−^. During the experiment, a certain amount of potassium dichromate (K_2_Cr_2_O_7_, ≥99.5%) was added into the groundwater to synthesis the Cr contaminated groundwater. All chemicals and reagents used were of analytical grade.

### 3.2. Nano-Magnetite Modified Biochar: Fabrication and Characterisation

The reed was initially washed by ultra-pure water, air-dried and then crushed into the powder. The powder was pyrolysed at 600 °C for 30 min under oxygen-limited conditions, with the N_2_ flow rate of 16.6 mL/min. After cooling to the room temperature, the pyrolysed product was passed through a 0.5 mm sieve. The pristine reed-derived biochar was then achieved after washing by ultra-pure water and dried in a vacuum oven for 24 h at 60 °C.

A ferrous sulphate solution was prepared by placing 1.0 ± 0.001 g of FeSO_4_·7H_2_O in 100 mL of ultra-pure water. The ultra-pure water was generated from the normal ultra-pure water generator (Millipore, Billerica, MA, USA) and the conductivity of water is 18.2 MΩ·cm at 25 °C. Then, 2.0 ± 0.001 g of reed biochar was added into the solution. The pH of the mixed biochar/Fe^2+^ was adjusted to 12 using 1 M NaOH solution, and extra NaOH (1 M) was added slowly with constant stirring until the Fe(OH)_3_ precipitate was formed. The solution was transferred to a custom-made microwave oven (700 W, 2450 MHz) under the treatment for 10 min. The solution was then cool down to ambient temperature and washed repeatedly with ultra-pure water until the pH became neutral. The remaining solids were separated from the solution and dried in a vacuum at 50–60 °C for 48 h. The final nano-magnetite modified biochar (m-biochar) was then achieved for the experiment.

The surface morphological characteristics and surface elemental compositions of both pristine biochar and m-biochar were analysed by scanning electron microscopy (SEM-EDS, Hitachi SU8020, Hitachi, Tokyo, Japan). The material was loaded with electrical tape, and after being sprayed with gold, the micro-area components were analysed. X-ray diffraction (XRD, D8 ADVANCE, BRUCKER, Bremen, Germany) was used to obtain the crystal structure and phase composition of m-biochar. Brunner–Emmet–Teller (BET) was used to measure the specific surface area, pore volume and pore size of the materials. The scanning range (2θ angle) was 10–90°, the scanning rate was 6°/min, and the scanning step was 0.02°. The surface functional group composition was determined by FTIR (Nicolet IS10, KBr plates, Thermofisher, Shanghai, China). The scanning step was 1 cm^−1^ and the range was from 400–4000 cm^−1^. X-ray photoelectron spectroscopy (XPS) analysed the changes of the valence of each element before and after Cr(VI) adsorption by m-biochar. The zeta potentials at different pH conditions were measured using a Malvern laser particle size analyser (Zetasizer Nano ZS, Malvern, UK). The determination of Cr(VI) concentrations in water were carried out by the diphenylcarbazide-based spectrophotometric method.

### 3.3. Batch Experiments

The preliminary test has been carried out to identify the appropriate ratio of dosage of biochar and Cr(VI) concentration (2:1), which could achieve 80% of the Cr(VI) adsorption. Then, a series of batch experiments were conducted to understand the Cr(VI) adsorption performances, kinetics and isotherms by the pristine biochar and m-biochar. For the kinetics experiment, 0.020 ± 0.001 g of biochar or m-biochar were added in a conical vessel, separately, which contained 10 mL of Cr(VI) solution. Three initial concentrations of Cr(VI) were set at 20.0 ± 0.1, 40.0 ± 0.1 and 80.0 ± 0.1 mg/L, respectively. The pH of each solution was adjusted to 5 prior to the experiment. During the adsorption experiment, the solution was placed in a temperature-controlled water bath shaker at 150 rpm under 25 ± 0.05 °C for 7 d. During the experiment, water samples (2 ± 0.02 mL) were collected at 0.5, 2, 12, 24, 48, 72, 96 and 120 h, respectively. The samples were filtered through a 0.22 μm mixed cellulose membrane to obtain a supernatant and subjected to Cr(VI) measurement.

Regarding the adsorption isotherm study, 0.020 ± 0.001 g of the pristine biochar and m-biochar were added separately into the Cr(VI) solutions (10 mL) with different initial concentrations, i.e., 10.0 ± 0.1, 20.0 ± 0.1, 30.0 ± 0.1, 40.0 ± 0.1, 50.0 ± 0.1, 60.0 ± 0.1, 70.0 ± 0.1 and 80.0 ± 0.1 mg/L in a batch of conical vessels. The solutions were continuously stirred at 150 rpm under 25 ± 0.05 °C for 7 d. The water samples were collected from each group for Cr(VI) detection along with the experiment. To investigate the effect of pH on the adsorption removal efficiency, initial Cr(VI) concentration of 20 ± 0.1 mg/L and 0.020 ± 0.001 g adsorbents were used for the experiment. Before the adsorption, the pH of each solution was set to 3, 5, 7, 9 and 11 using 1 M HCl or NaOH solutions. The experimental conditions were the same as those in the kinetic experiment. During all the adsorption experiments, the blank control group was set without any adsorbent addition. Each experimental group and samples were carried out in triplicate.

### 3.4. Calculations

When the adsorption reactions reached the equilibrium, the adsorption capacity (*q_e_*, mg/g) was calculated by Equation (3),

(3) $$q_{e}=\frac{(c_{0}—c_{e})V}{m}$$

where *q_e_* is the equilibrium adsorption amount of Cr(VI) in the adsorbent, mg/g; *C*_0_ and *C_e_* are the mass concentration of Cr(VI) in groundwater at the initial time and the equilibrium time, respectively, mg/L; *V* is the volume of groundwater (L) and m is the mass of the adsorbent (g).

The adsorption kinetics of Cr(VI) on both pristine biochar and m-biochar were simulated using a pseudo-first-order kinetic model (Equation (4)), a pseudo-second-order kinetic model (Equation (5)) and an intra-particle diffusion model (Equation (6)).

(4) $$\ln(q_{e}-q_{t})=\ln q_{e}-k_{1}t$$

(5) $$\frac{t}{q_{t}}=\frac{1}{k_{2}q_{e}^{2}}+\frac{t}{q_{e}}$$

(6) $$q_{t}=k_{d}t^{0.5}+C$$

where *q_t_* is the adsorption amount of Cr(VI) per unit mass m-biochar when the time *t* (h) elapses, mg/g; *k*_1_ is the pseudo-first-order kinetic adsorption rate constant, 1/h; *k*_2_ is the pseudo-second-order kinetic adsorption rate constant, g/(mg·h); *k_d_* is the intraparticle diffusion rate constant, [mg/(g·h^0.5^)].

At constant temperature, the relationships between equilibrium adsorption concentrations of Cr(VI) were simulated using the Langmuir adsorption isotherm equation (Equation (7)) and Freundlich adsorption isotherm equation (Equation (8)).

(7) $$\frac{c_{e}}{q_{e}}=\frac{1}{q_{m}k_{L}}+\frac{c_{e}}{q_{m}}$$

(8) $$\ln q_{e}=\frac{1}{n}\ln C_{e}+\ln K_{f}$$

where *q_m_* is the saturated adsorption amount of Cr(VI) on m-biochar, mg/g; *K_L_* is a constant related to the adsorption performance. *K_f_* is the Freundlich adsorption coefficient, (μg/g)/(μg/L)^n^; *n* is Freundlich adsorption linear index.

### 3.5. Statistical Analysis

SigmaPlot software (version 12.5, Systat Software Inc., San Jose, CA, USA) was used for plotting and data analyses. One-way analysis of variance (ANOVA) was used to evaluate the significant difference of the Cr(VI) adsorption between different groups under different conditions of pH, temperature, response time and Cr(VI) Initial concentrations (*p <* 0.05).

## 4. Conclusions

Nano-magnetite modified biochar (m-biochar) was prepared by reed as the raw material through a simple and rapid in situ microwave synthesis method in this study. The results of the FTIR and XRD characterisations supported that nano-Fe_3_O_4_ has been successfully loaded on the biochar and altered its pore structure. The synthesised m-biochar achieved enhanced performance for Cr(VI) removal from contaminated groundwater compared to the pristine biochar. Cr(VI) adsorption followed a pseudo-second-order model and fitted to an intraparticle diffusion model indicated that particle diffusion process governed the Cr(VI) adsorption rate. Moreover, the pH was demonstrated as an important factor, where the relatively lower pH conditions were more conducive to the removal of Cr(VI) in groundwater. Under acidic conditions, the carbon-containing functional groups of m-biochar had a stronger ability to undergo redox reactions with Cr(VI). This study has highlighted a potential, effective synthesis method to prepare a superior Cr(VI) adsorbent, however, the adsorbent performance improvement, as well as the up-scale demonstration and application, still needs further studies.

## Figures and Tables

**Table 1:** Physical characteristics of the pristine biochar and m-biochar.

Materials	BET Surface Area (m^2^/g)	Total Pore Volume (cm^3^/g)	Micropore Volume (cm^3^/g)
biochar	51.23	0.02	0.01
m-biochar	154.79	0.09	0.05

**Table 2:** The kinetic parameters for Cr(VI) adsorption on the pristine biochar and m-biochar.

Cr (VI) (mg/L)	Materials	Pseudo-First-Order Kinetics			Pseudo-Second-Order Kinetics			Intra-Particle Diffusion Model					
		*q_e_* (mg/g)	*k*_1_ (h^−1^)	*R* ^2^	*q_e_* (mg/g)	*k*_1_ [g/(mg·h)]	*R* ^2^	*k*_d1_ [mg/(g·h^0.5^)]	*C_1_*	*R*_1_^2^	*k*_d2_ [mg/(g·h^0.5^)]	*C*_2_	*R*_2_^2^
20	biochar	5.40	0.08	0.86	5.55	0.04	0.92	0.56	1.34	1.00	0.12	4.38	0.66
	m-biochar	5.83	0.44	0.86	6.22	0.08	0.92	0.66	1.97	1.00	0.13	5.19	0.73
40	biochar	5.60	0.45	0.85	6.02	0.08	0.92	0.63	1.79	0.94	0.13	5.04	0.49
	m-biochar	6.47	0.59	0.88	6.74	0.12	0.94	0.72	2.52	0.97	0.06	6.41	0.80
80	biochar	6.38	0.32	0.92	6.94	0.05	0.97	1.00	1.05	0.85	0.12	5.82	0.62
	m-biochar	7.16	0.63	0.86	7.46	0.12	0.92	0.70	3.05	0.96	0.12	6.74	0.75

**Table 3:** Isotherm parameters for Cr(VI) adsorption on pristine biochar and m-biochar.

Materials	Langmuir			Freundlich		
	*q_m_* (mg/g)	*k*_L_ (L/mg)	*R* ^2^	*n*	*k*_f_ (μg/g)/(μg/L)^n^	*R* ^2^
biochar	6.81	0.86	0.90	0.13	4.07	0.98
m-biochar	7.72	0.77	0.95	0.14	4.48	0.92

**Table 4:** Zeta Potential of different biochar adsorbents under different pH conditions.

pH	Adsorbents	Zeta Potential	Reference
2	Iron/zinc Biochar(Fe@Zn@HBC)	42.9	[12]
2	ZVI Magnetic Biochar(FeBC_800_)	~23	[18]
2–4	MPHC-HDA	>0	[19]
3	Palm fiber biochar(BC)	~5	[39]
3	MBCO ^a^	~15	[39]
Below 4.2	N-doped magnetic agar biochar (ABF-N_800_)	>0	[40]
4.61	Magnetic Biochar (SMBC2)	45.7	[17]
7–11	NiAl layered double oxides modified magnetic corncob biochar	<0	[41]
10.11	Pennisetum hydridum biochar	−48.1	[42]
3	biochar	7.99	This paper
5	biochar	3.72	This paper
7–11	biochar	<0	This paper
3	m-biochar	26.7	This paper
5	m-biochar	19.7	This paper
7–11	m-biochar	<0	This paper

^a^ The palm fibre biochar was oxidized by hydrogen peroxide (BCO) and loaded of Fe_3_O_4_ nano-particles.
